# Synergistic anti-proliferative effects of gambogic acid with docetaxel in gastrointestinal cancer cell lines

**DOI:** 10.1186/1472-6882-12-58

**Published:** 2012-04-30

**Authors:** Zhengyun Zou, Li Xie, Jia Wei, Lixia Yu, Xiaoping Qian, Junhao Chen, Tingting Wang, Baorui Liu

**Affiliations:** 1 The Comprehensive Cancer Center of Drum Tower Hospital, Medical School of Nanjing University & Clinical Cancer Institute of Nanjing University, Nanjing, 210008, China; 2 Department of Laboratory, Drum Tower Hospital, Medical School of Nanjing University, Nanjing, 210008, China; 3 Medical School of Nanjing University, Nanjing, 210008, China

## Abstract

**Summary:**

**Background**

Gambogic acid has a marked anti-tumor effect for gastric and colorectal cancers *in vitro* and *in vivo*. However, recent investigations on gambogic acid have focused mainly on mono-drug therapy, and its potential role in cancer therapy has not been comprehensively illustrated. This study aimed to assess the interaction between gambogic acid and docetaxel on human gastrointestinal cancer cells and to investigate the mechanism of gambogic acid plus docetaxel treatment-induced apoptotic cell death.

**Methods**

MTT assay was used to determine IC_50_ values in BGC-823, MKN-28, LOVO and SW-116 cells after gambogic acid and docetaxel administration. Median effect analysis was applied for determination of synergism and antagonism. Synergistic interaction between gambogic acid and docetaxel was evaluated using the combination index (CI) method. Furthermore, cellular apoptosis was analyzed by Annexin-V and propidium iodide (PI) double staining. Additionally, mRNA expression of drug-associated genes, i.e., β-tublin III and tau, and the apoptosis-related gene survivin, were measured by quantitative reverse transcription polymerase chain reaction (qRT-PCR).

**Results**

Gambogic acid provided a synergistic effect on the cytotoxicity induced by docetaxel in all four cell lines. The combined application of gambogic acid and docetaxel enhanced apoptosis in gastrointestinal cancer cells. Moreover, gambogic acid markedly decreased the mRNA expression of docetaxel-related genes, including β-tubulin III, tau and survivin, in BGC-823 cells.

**Conclusions**

Gambogic acid plus docetaxel produced a synergistic anti-tumor effect in gastrointestinal cancer cells, suggesting that the drug combination may offer a novel treatment option for patients with gastric and colorectal cancers.

## Background

Despite a decline in incidence, gastric cancer remains one of the leading causes of cancer-related deaths worldwide [1]. Colorectal cancer is the third most common cancer worldwide, with an incidence of approximately one million cases and 500,000 mortalities annually [2,3]. Despite recent advances in chemotherapeutic treatment of gastric cancer, no single agent or combination regimen has been accepted as a standard therapy [4]. For colorectal cancer, capecitabine monotherapy or combination chemotherapies such as FOLFOX (infusional 5-fluorouracil/leucovorin with oxaliplatin) and FOLFIRI (infusional 5-fluorouracil/leucovorin with irinotecan) are considered standard regimens. However, the positive response rate is still lower than 40% [5,6]. Hence, better systemic therapies are needed to decrease side effects and improve the clinical outcomes of patients with gastric and colorectal cancer.

Docetaxel (Doc), a widely used taxane for the treatment of a variety of cancers, shares a similar mechanism of action with paclitaxel [7]. Emerging lines of evidence indicate that Doc is quite active against several human malignancies, including gastric cancer [8,9]. Nevertheless, a poor prognosis has been observed after Doc monotherapy. A previous report demonstrated that administration of 100 mg/m^2^ Doc to patients with adenocarcinoma of the upper gastrointestinal tract, previously untreated with cytotoxic chemotherapy, yielded a 17% response rate and only 2.8 months of median time of progression (TTP)[9]. It has been reported that Doc plus cisplatin induces an overall response rate of 37.2% in patients with advanced gastric cancer, [10] suggesting that Doc-containing combination therapy might be useful in the management of gastric cancer.

Our previous study demonstrated that gambogic acid (GA), the primary active component of gamboge, reversed Doc resistance in BGC-823/Doc gastric cancer cells [11]. Moreover, GA alone or in combination with Doc significantly down-regulates the mRNA expression of survivin in gastric cancer cells, [11] implying that GA may promote the anti-tumor effect of Doc through promotion of apoptotic cell death. However, the synergistic anti-tumor effect of GA and Doc in gastrointestinal cancer cells has not yet been clearly illustrated.

In the present study, two gastric cancer cell lines, MKN-28 and BGC-823, and two colorectal cancer cell lines, LOVO and SW-116, were used to examine the effects of GA and Doc mono-therapy or combination therapy. Our results indicated that administration of GA plus Doc enhanced apoptotic cell death in gastrointestinal cancer cells compared to a single drug application, suggesting that this drug combination may provide a novel and beneficial treatment option for patients with gastric and colorectal cancers.

## Methods

### Reagents

Gambogic acid (GA, molecular formula C_38_H_44_O_8_) was provided by Jiangsu Kangyuan Pharmaceutical Co., Ltd. (Jiangsu, China). Docetaxel (Doc) was obtained from Jiangsu Hengrui Pharmaceutical Co., Ltd. (Jiangsu, China). GA and Doc were prepared in complete culture medium immediately prior to use. RPMI 1640 medium was purchased from GIBCO BRL (Grand Island, NY, USA). Fetal bovine serum was obtained from Lanzhou National Hyclone Bio-engineering Co., Ltd. (Lanzhou, Gansu, China). MTT (3-[4, 5-dimethylthiazol-2-yl]-2, 5-diphenyltetrazolium bromide) was purchased from Sigma Chemical Co. (St. Louis, MO, USA). Dimethyl sulfoxide (DMSO) was provided by Shanghai Ling-feng Chemical Reagents Co. (Shanghai, China). All other chemicals used in this study were of the highest purity available.

### Cell culture

Human cancer cell lines, specifically the gastric cancer cell lines MKN-28 and BGC-823 and the colorectal cancer cell lines LOVO and SW-116, were obtained from Shanghai Institute of Cell Biology (Shanghai, China). Cells were maintained in RPMI 1640 medium supplemented with 10% fetal bovine serum. Cultures were incubated at 37°C in a humidified atmosphere of 5% CO_2_.

### Evaluation of cytotoxicity

Cytotoxicity was measured using a MTT assay. Briefly, tumor cells in log-phase were trypsinized and seeded at a density of 2 × 10^3^ cells per well onto 96-well plates. After 24 h, cells were treated with GA, Doc, or GA plus Doc at different concentrations. MTT (1/10 volume) was added to each well after 48-h drug treatments, and plates were further incubated at 37°C for another 4 h. Formazan crystals formed were dissolved in DMSO. Absorbance (OD) was determined with a multiwell spectrophotometer (BioTek, Winooski, VT, USA) at 570 nm. Absorbance values were expressed as percentages relative to untreated controls. The IC_50_ was defined as the concentration required for 50% inhibition of cell growth. Each condition was tested in quintuplicate, and at least three independent experiments were performed. The inhibition rate was calculated according to the following equation: Inhibition rate (IR) (%) = [(average OD value in the control group - average OD value in the treatment group) / average OD value in the control group] × 100%.

### Determination of synergism and antagonism

Synergism or antagonism after drug treatments was quantitated with the median-effect principle, using the combination index (CI) method [12]. The CI is defined by the following equation: CI = [(D)1 / (Dx)1] + [(D)2 / (Dx)2] + [α(D)1(D)2 / (Dx)1(Dx)2], where (Dx)1 and (Dx)2 are the concentrations for D1 (GA) and D2 (Doc) that give x% inhibition, whereas (D)1 and (D)2 in the numerators are the concentrations of the two drugs that produce an identical level of effect in combination. The parameter α = 0 when the drugs are mutually exclusive (i.e., with similar modes of action), while α = 1 if they are mutually non-exclusive (i.e., with independent modes of action). CI values > 1 indicate antagonism, CI values < 1 indicate synergy, and CI values = 1 indicate additivity. Each CI ratio represented here is the mean value derived from at least three independent experiments.

### Assessment of apoptotic and necrotic cell death

Apoptotic or necrotic cell death was evaluated using an annexin V-fluorescein isothiocyanate (FITC) Apoptosis Detection Kit (BioVision, Mountain View, CA, USA) according to the manufacturer’s instructions. MNK-28 cells were treated with 0.25 μM GA and 0.625 μM Doc; BGC-823 cells were treated with 2.5 μM GA and 6.25 μM Doc; LOVO and SW-116 cells were treated with 1 μM GA and 2.5 μM Doc. After 48 h of drug treatment, cells were harvested and resuspended in 500 μL of binding buffer. Annexin V-FITC and propidium iodide (PI) were then added. Percentages of apoptotic or necrotic cells were analyzed with a FACScan flow cytometer (Becton Dickinson, Sunnyvale, CA, USA). The cells in the annexin V^+^ and PI^–^ fraction were identified as early apoptotic cells, while those in the annexin V^+^ and PI^+^ fraction represented late apoptotic cells or necrotic cells.

### Quantitative reverse transcription polymerase chain reaction (qRT-PCR)

Total RNA was extracted from BGC-823 cells after 48 h incubation with or without GA. cDNA was synthesized using random primers and Primescript reverse transcriptase (Takara; Shiga, Japan). qPCR reactions for indicated genes were carried out using SYBR green qPCR kit (Takara; Shiga, Japan) by a fluorescent temperature cycler (Mx3000P Real Time PCR System; Stratagene; Santa Clara, CA, USA). Sequences of primers (forward and reverse, respectively) were as follows: β-tubulin III: 5’-AGCAAGAACAGCAGCTACTTCGT-3’ and 5’-GATGAAGGTGGAGGACATCTTGA-3’; tau: 5’- GATTGGGTCCCTGGACAATA-3’ and 5’-GTGGTCTGTCTTGGCTTTGG-3’; survivin: 5’-ATTCGTCCGGTTGCGCTTTCC-3’ and 5’-CACGGCGCACTTTCTTCGCAG-3’; β-actin: 5’-GCGAGAAGATGACCCAGATC-3’ and 5’-GGATAGCAACGCCTGGATAG-3’. Cycling conditions were as follows: denaturation (95°C for 10 sec), amplification and quantitation repeated for 45 cycles (95°C for 5 s, 60°C for 20 s, with a single fluorescence measurement); a melting curve program (95°C for 10 s, 55-95°C with a heating rate of 0.1°C/s and continuous fluorescence measurement). Relative gene expression was quantified according to the comparative Ct method using β-actin as an internal standard and cells without GA treatment as calibrators. Gene expression was analyzed with the Stratagene analysis software and quantified by the 2^-ΔΔCt^ method.

### Statistical analysis

Data were analyzed by using SPSS 15.0 software and were expressed as means ± SD. Statistical analysis was performed using the Student’s *t*-test. Values of *P* < 0.05 were considered to be statistically significant.

## Results

### Cytotoxic effects of GA and Doc treatment on cancer cells

**Figure 1:**
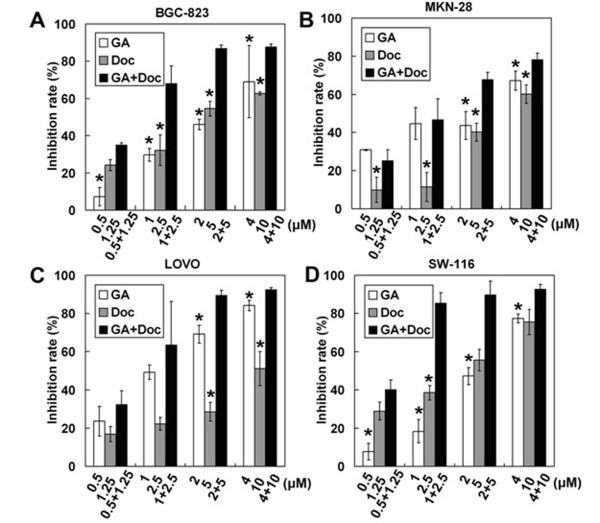
**Cytotoxic effects of GA and Doc treatment in cancer cells.** After 48 h incubation with GA, Doc or GA plus Doc at the indicated concentrations, cytotoxicity was determined by the MTT assay. Inhibition rates (%) in BGC-823 (**A**), MKN-28 (**B**), LOVO (**C**) and SW-116 (**D**) cells were calculated as described in Methods. *P < 0.05 compared with GA plus Doc.

The cytotoxic effects of GA and Doc on cancer cells were first evaluated by using the MTT assay. As expected, GA or Doc alone dose-dependently increased cytotoxicity in the gastric cancer cell lines MKN-28 and BGC-823 and the colorectal cancer cell lines LOVO and SW-116 (Figure 1). Combined application of GA and Doc markedly enhanced the cytotoxic effects in each of the cell lines as compared with single drug administration (*P* < 0.05). Table 1 shows the half-maximal inhibitory concentration (IC_50_) values for four cancer cells lines exposed to GA or Doc. It should be noted that the lowest IC_50_ for GA or Doc treatment was detected in LOVO (1.21 ± 0.11 μM) and SW-116 (1.77 ± 0.43 μM) cells, respectively. We then used fixed-ratio concentrations including each drug IC_50_ dose for the remaining experiments.

**Table 1:** **IC**_50_** value (μM) of different cells after 48 h of GA or Doc treatment**

		Cell line	GA	Doc
		BGC-823	2.33 ± 0.05	4.93 ± 0.57
		MKN-28	1.66 ± 0.11	8.63 ± 1.90
		LOVO	1.21 ± 0.11	4.49 ± 0.74
		SW-116	2.14 ± 0.33	1.77 ± 0.43

## Synergistic effects of GA and Doc on gastrointestinal cancer cells

**Figure 2:**
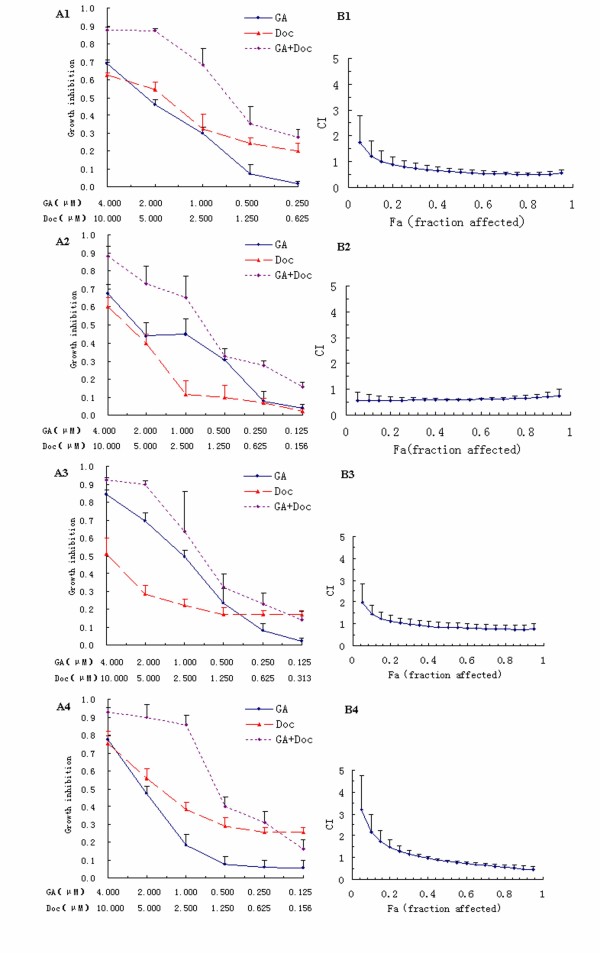
**Synergistic effects of GA and Doc on gastrointestinal cancer cells.** (**A1-4**): Dose–response curves for BGC-823 (A1), MKN-28 (A2), LOVO (A3) and SW116 (A4) cells. (**B1-4**): CI values at different levels of growth inhibitory effect (the fraction of cells affected, Fa) for BGC-823 (B1), MKN28 (B2), LOVO (B3) and SW116 (B4) cells.

To investigate whether GA could enhance the anti-tumor effects of Doc, which is widely used in cancer chemotherapy, the effects of each drug (administered alone or in combination) were examined. The concentration ratio for the combined application of GA and Doc was set as 1: 2.5, which is approximately the same as the ratio of the IC_50_ values for each drug. Figure 2 shows the dose–response curves and CI values at different levels of growth inhibition effect (fraction affected, Fa) when GA and Doc were administrated alone or in combination to gastrointestinal cancer cells. In the gastric cancer cell lines MKN-28 and BGC-823 and the colorectal cancer cell lines LOVO and SW-116, the combination treatment provided an efficient anti-tumor response compared with single drug application, as revealed by the dose–response curves (Figure 2A1-4).

CI values at different levels of Fa demonstrated that when Fa > 0.15 for BGC-823 cells, > 0.25 for LOVO cells, and > 0.40 for SW-116 cells, the CI values were < 1, and the combination treatment with GA and Doc could induce a synergistic, anti-proliferative effect (Figure 2B1-4). The CI value was < 1 across almost the entire dose-inhibition range when GA was combined with Doc in MKN-28 cells, indicating a more pronounced synergistic effect for the combination therapy. These results are summarized in Table 2, which indicates for each combination, a computer-calculated CI for 20%, 40%, 60% and 80% cytotoxicity (Fa = 0.2, 0.4, 0.6, 0.8, respectively).

**Table 2:** Summary of CI values at 20%, 40%, 60% and 80% fraction affected

	CI (means ± SD)			
Cell line	20%	40%	60%	80%
BGC-823	0.87 ± 0.31	0.64 ± 0.15	0.54 ± 0.09	0.50 ± 0.07
MKN-28	0.57 ± 0.14	0.59 ± 0.06	0.61 ± 0.03	0.65 ± 0.11
LOVO	1.11 ± 0.26	0.90 ± 0.23	0.80 ± 0.21	0.76 ± 0.18
SW-116	1.47 ± 0.34	0.97 ± 0.07	0.73 ± 0.08	0.56 ± 0.14

### Combination treatment enhances apoptotic cell death

**Figure 3:**
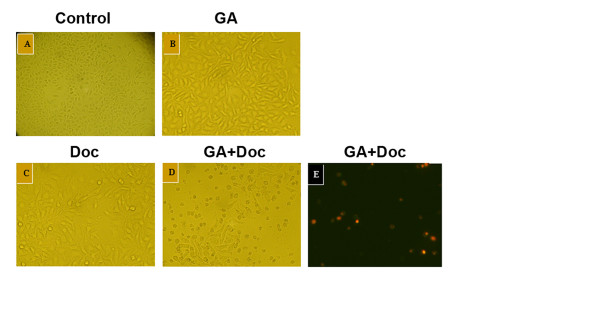
**Induction of MKN-28 cell death by GA and Doc.** After 48 h incubation with GA (0.25 μM) (**b**), Doc (0.625 μM) (**c**) or GA (0.25 μM) plus Doc (0.625 μM) (**d**), morphological changes in MKN-28 cells were observed under light microscopy. Cells without any treatments were used as controls (**a**). (**e**) Annexin-V and PI double staining for cells from panel **d**). Samples were visualized under a fluorescent microscope. Annexin-V-labeled cells with green fluorescence were recognized as early apoptotic cells; annexin-V and PI double-labeled cells with orange fluorescence were recognized as late apoptotic or necrotic cells.

**Figure 4:**
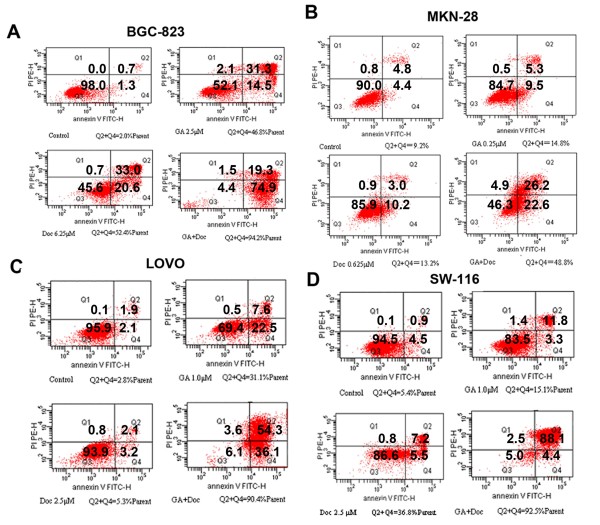
**Induction of apoptotic cell death by GA and Doc.** After 48 h incubation with GA, Doc or GA plus Doc at the indicated concentrations, cells were stained with annexin-V and PI as described in Methods. Apoptosis of BGC-823 (**A**), MKN-28 (**B**), LOVO (**C**) and SW-116 (**D**) cells was analyzed by flow cytometry.

Based on the results obtained with the MTT assay and the middle-effect principle, the lower concentration of GA plus Doc could also induce remarkable growth suppression in gastrointestinal cancer cells. Hence, 0.25 μM GA and 0.625 μM Doc were administered for evaluation of apoptotic cell death in MKN-28 cells. Forty-eight hours after drug incubation, the morphological alterations of MKN-28 cells were observed using light microscopy. As shown by Figure 3, increased cell loss was found after GA (0.25 μM) plus Doc (0.625 μM) treatment. Similar with the results obtained from MKN-28 cells, Annexin-V and PI double staining of the other three cell lines all revealed that the combination treatment induced apoptotic cell death (data not shown). Apoptosis of BGC-823, MKN-28, LOVO and SW-116 cells after combination treatment was further determined by flow cytometry. Consistent with previous observations, the proportion of apoptotic cells (annexin V^+^ PI^+/−^) was markedly increased by application of GA plus Doc in all four cell lines as compared with GA or Doc treatment alone (Figure 4). These results indicate that the combination treatment enhanced apoptotic cell death in gastrointestinal cancer cells.

### GA downregulates the expression of genes involved in docetaxel sensitivity

**Figure 5:**
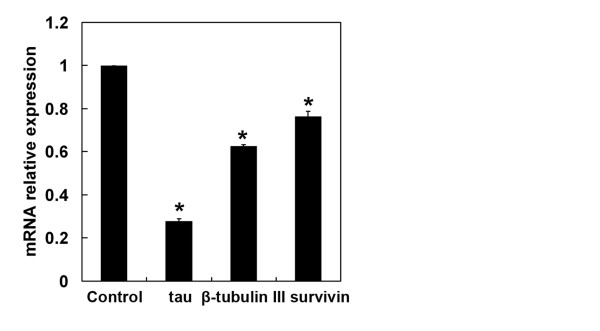
**GA down-regulates the expression of genes involved in Doc sensitivity**. The mRNA expression of genes involved in Doc sensitivity, including β-tubulin III, tau and survivin, in BGC-823 cells was determined by qRT-PCR after 48 h of GA (0.25 μM) treatment. The mRNA expression of target genes was normalized to a control. *P < 0.01 compared with control. Data were quantified from three independent experiments.

Accumulating evidence indicates that various cellular proteins that are associated with microtubules can determine the sensitivity of cancer cells to microtubule-targeting agents and play a role in tumor cell resistance to these agents [13]. To explore the potential mechanism by which GA plus Doc induces apoptotic cell death, the expression of genes involved in Doc sensitivity that regulate microtubules, such as class III β-tubulin (β-tubulin III) and tau, were evaluated by qRT-PCR. It should be noted that 48 h of GA (0.25 μM) incubation markedly down-regulated the expression of β-tubulin III and tau (Figure 5). Moreover, the expression of survivin, which is recognized as an apoptosis inhibitor, [14] was also suppressed by GA administration (Figure 5). These results imply that β-tubulin III, tau and survivin might be involved in the GA-mediated anti-tumor effect.

## Discussion

Gastrointestinal cancer is the most commonly diagnosed cancer worldwide, exhibiting a high annual mortality [1-3]. Chemotherapy has been recognized as the treatment of choice for patients with gastric or colorectal cancer, either alone or in combination with surgery with or without radiotherapy [15,16]. Various chemotherapeutic agents have been used for clinical treatment of patients, including mitomycin C, cisplatin, doxorubicin, 5-fluorouracil (5-FU), paclitaxel and Doc [17]. It has been reported that the response rates range from 10% to 30% for single-agent therapy and 30% to 60% for combination therapy [17]. However, the overall outcome remains poor due to local or distant recurrences, drug resistance or side effects derived from the various drugs. Accumulating evidence suggests that certain natural phytochemical compounds used in conjunction with chemotherapeutic agents could enhance therapeutic efficacy by sensitizing cells to treatment [18].

Doc, which is widely used for the treatment of several human malignancies, has been reported to be active against gastric cancer [8,9]. However, administration of Doc alone often leads to undesirable side effects and drug resistance [9,16]. The development of new taxane anticancer agents with fewer side effects, superior pharmacological properties and improved activity against drug-resistant human cancers seems to be a critical need for treatment of gastric cancer [19]. Moreover, Doc-containing chemotherapy has gradually developed with the aim of improving overall response rate and survival [10].

GA, the primary active component of gamboge, has been reported to induce apoptotic cell death of human gastric cancer SGC-7901 and BGC-823 cells [20,21]. Treatment of Doc resistant BGC-823/Doc cells with GA led to a dramatic increase in Doc-induced cytotoxicity. Cell cycle analysis further indicated that GA treatment potentiated Doc-induced G2/M cell cycle arrest. Moreover, GA singly or in combination with Doc significantly downregulated the mRNA expression of survivin [11]. In addition, combined treatment of GA and 5-FU caused significant growth inhibition of BGC-823 human tumor xenografts in vivo [22] Our previous study demonstrated that GA reversed Doc resistance in BGC-823/Doc gastric cancer cells [11]. In the present study, the combined application of GA and Doc exhibited synergistic and efficient anti-tumor effects in the human gastric cancer cell lines MKN-28 and BGC-823, and colorectal cancer cell lines LOVO and SW-116 (Figures 1 and 2), suggesting that GA may enhance the anti-cancer effects of Doc in both gastric and colorectal cancers. In addition, combination therapy suppressed the proliferation of cancer cells in a dose-dependent manner (Figure 1). Furthermore, the colorectal cancer cell line LOVO exhibited the lowest IC_50_ following GA administration, followed by the highly differentiated human gastric cancer cell MKN-28. With regard to Doc treatment, SW-116 cells were the most sensitive cell line followed by BGC-823 cells. However, combined administration of GA and Doc provided a synergistic effect on growth suppression of MKN-28 cells. Based on these observations, we hypothesize that growth of highly differentiated tumor cells, such as MKN-28 cells, could be synergistically suppressed by GA and Doc treatment. These observations are in accordance with previous reports showing that the combined application of 5-FU and GA had a stronger anti-gastric cancer effect in BGC-823 cells than that of 5-FU or GA alone [22].

Survivin, a novel inhibitor of apoptosis (IAP), has been reported to be associated with apoptosis, proliferation and angiogenesis during human colorectal tumorigenesis [23]. Moreover, survivin is also involved in tumor cell resistance to certain anti-cancer agents [24]. Our previous study demonstrated that GA reversed Doc resistance in BGC-823/Doc gastric cancer cells by down-regulating the expression of survivin,[11] implying that GA may promote the anti-tumor effect of Doc through mediation of apoptotic cell death. Here, we found that combination therapy significantly enhanced cell apoptosis (Figures 3 and 4). In addition, GA reduced the mRNA expression of survivin, suggesting that apoptosis might be involved in the GA-mediated anti-tumor effect.

Microtubule-associated proteins, such as β-tubulin III and tau, are known as essential predictive markers for the sensitivity of taxane-based chemotherapy in several human cancers [25,26]. Reduced expression of microtubule-associated proteins is linked to better outcomes for taxane therapy. Doc binds to β-tubulin, which is one of the major components of microtubules, and exerts its growth-inhibitory effects through G2/M cell cycle arrest. This then induces mitochondrial dysfunction and cell apoptosis. In the present study, GA treatment markedly decreased the mRNA expression of β-tubulin III and tau, suggesting these two genes were also involved in the GA-induced anti-tumor effect.

In summary, our present study demonstrates that GA and Doc combination treatment induced a synergistic, anti-tumor effect in gastric and colorectal cancer cells. Future studies will be focused on exploring the mechanism of the interaction of GA and Doc in gastrointestinal cancer cells and investigating the combination therapy *in vivo* using animal models.

## Competing interests

The authors declare that they have no competing interests.

## Authors’ contributions

Zhengyun Zou designed the study protocol, performed synergistic experiments and prepared the manuscript. Li Xie, Jia Wei, LixiaYu and Tingting Wang performed qRT-PCR. Xiaoping Qian performed statistical analysis. Junhao Chen analyzed apoptosis. Baorui Liu designed the study protocol together with the first author. All authors read and approved the final manuscript.

## Pre-publication history

The pre-publication history for this paper can be accessed here:

http://www.biomedcentral.com/1472-6882/12/58/prepub
